# Identification of gene signatures from RNA-seq data using Pareto-optimal cluster algorithm

**DOI:** 10.1186/s12918-018-0650-2

**Published:** 2018-12-21

**Authors:** Saurav Mallik, Zhongming Zhao

**Affiliations:** 1 Center for Precision Health, School of Biomedical Informatics, The University of Texas Health Science Center at Houston, Houston, 77030 TX USA; 2 Department of Biomedical Informatics, Vanderbilt University School of Medicine, Nashville, 37232 TN USA

**Keywords:** Gene signature, Cervical cancer, Pareto optimal clustering, K-means, Limma

## Abstract

**Background:**

Gene signatures are important to represent the molecular changes in the disease genomes or the cells in specific conditions, and have been often used to separate samples into different groups for better research or clinical treatment. While many methods and applications have been available in literature, there still lack powerful ones that can take account of the complex data and detect the most informative signatures.

**Methods:**

In this article, we present a new framework for identifying gene signatures using Pareto-optimal cluster size identification for RNA-seq data. We first performed pre-filtering steps and normalization, then utilized the empirical Bayes test in Limma package to identify the differentially expressed genes (*DEG*s). Next, we used a multi-objective optimization technique, “Multi-objective optimization for collecting cluster alternatives” (MOCCA in R package) on these *DEG*s to find Pareto-optimal cluster size, and then applied k-means clustering to the RNA-seq data based on the optimal cluster size. The best cluster was obtained through computing the average Spearman’s Correlation Score among all the genes in pair-wise manner belonging to the module. The best cluster is treated as the signature for the respective disease or cellular condition.

**Results:**

We applied our framework to a cervical cancer RNA-seq dataset, which included 253 squamous cell carcinoma (SCC) samples and 22 adenocarcinoma (ADENO) samples. We identified a total of 582 *DEG*s by Limma analysis of SCC versus ADENO samples. Among them, 260 are up-regulated genes and 322 are down-regulated genes. Using MOCCA, we obtained seven Pareto-optimal clusters. The best cluster has a total of 35 *DEG*s consisting of all-upregulated genes. For validation, we ran PAMR (prediction analysis for microarrays) classifier on the selected best cluster, and assessed the classification performance. Our evaluation, measured by sensitivity, specificity, precision, and accuracy, showed high confidence.

**Conclusions:**

Our framework identified a multi-objective based cluster that is treated as a signature that can classify the disease and control group of samples with higher classification performance (accuracy 0.935) for the corresponding disease. Our method is useful to find signature for any RNA-seq or microarray data.

## Background

Detection of gene signatures from genomic data has been an important topic in medical domain during the last two decades. A “gene signature” can be stated as a single or a group of genes in a cell having a unique pattern of gene expression that is the consequence of either changed biological process or altered pathogenic medical terms.

Statistical analysis [1–7] is one of the most crucial techniques to determine differentially expressed transcripts [8–17] across a group of samples versus another group of samples. For RNA-seq data, proper selection of normalization and statistical test are very important, otherwise it might generate wrong *p*-value for each transcript. Voom normalization [18] is very useful for the RNA-seq data, whereas Limma tool [2, 19–21] is also useful for this kind of data.

Determining optimal cluster (module) number of the data is a challenging problem. In general, we set the number of clusters whenever we use any clustering algorithm. Thus, that is not optimal. Hence, as a result, this might increase the error-rate. Therefore, prior to use any clustering algorithm, it is necessary to estimate the Pareto-optimal cluster size using the combination of several clustering algorithms and various cluster validation indices as multi-objectives. It obviously reduces the error-rate whenever clustering on the underlying data. Of note, in case of the single-objective optimization problem [22], the superiority of a solution over other existing solutions can be produced very easily through the comparison of the scores of their objective functions. However, in the case of the multi-objective optimization problem [14, 15, 23–25] the goodness of a solution is generally identified through the dominance. The non-dominated solution set is basically a set of all the solutions which can not be dominated by none of the members of the solution set. Interestingly, the non-dominated set of the whole feasible decision space is stated as the Pareto-optimal set [26], whereas the boundary defined by the set of all the points that are mapped from the Pareto-optimal set, is denoted as the Pareto-optimal front [26]. MOCCA (Multi-objective optimization for collecting cluster alternatives) [27] is a latest robust estimator of Pareto-optimal cluster size through aggregating the best cluster numbers of various clustering algorithms and several cluster validation indices as the multi-objectives. MOCCA provides the ranking of the Pareto-optimal cluster sizes based upon their domination.

There are many bioinformatics approaches available for the gene signature identification. Mitra et al. identified gene-signature using a machine learning techniques including the Random forest and random survival forest algorithm for multiple myeloma [28]. Aziz et al. analyzed a microarray data using GeneSpring software and other existing R software and determined gene-signature in colorectal cancer [29]. Chen et al. applied a decision-tree analysis and survival analysis to identify gene signature on nonsmall-cell lung cancer [30]. Other related research works were [31–43]. So far, a very few attempts has been performed using through Pareto-optimal technique for gene signature identification. Basu et al. proposed Strength Pareto Evolutionary Algorithm for gene set selection [44]. Furthermore, neither pathway analysis nor gene-ontology analysis was carried out by them. Awad and Jong proposed another method of optimization of Spectral Signatures Selection through MOGA Multi-Objective Genetic Algorithm [45]. The overall performance of these methods are not so satisfactory. Hence, in this article, we developed a new framework of identifying gene signature using Pareto-optimal cluster identification for RNA-seq data. In this regard, we conducted some pre-filtering steps to remove the redundant feature from the dataset. Next, we utilized Voom normalization and then Limma tool to identify the differentially expressed genes. Thereafter, we applied MOCCA R tool on these differentially expressed genes using 12 objectives (i.e., kmeans.MCA, kmeans.Jaccard, kmeans.FM, kmeans.CQS, neuralgas.MCA, neuralgas.Jaccard, neuralgas.FM, neuralgas.CQS, single.MCA, single.Jaccard, single.FM and single.CQS) to estimate the Patero-optimal cluster size, and then applied k-means clustering through the optimal cluster size. The best cluster was obtained through computing the average Spearman’s Correlation Score among all the genes in pair-wise manner belonging to the module. The best cluster is now treated as a signature for the respective disease. There are many ways to validate the gene signature. One of them is classification of the features (genes) belonging to the signature. For the purpose of validation, we applied PAMR (prediction analysis for microarrays) [46] classifier on all the features (genes) of selected best cluster, and computed the classification performances. In this work, we used TCGA cervical cancer dataset for experiment. We obtained high classification accuracy in the performance of the classifier. Finally, our method is useful to find signatures for any RNA-seq or similar kind of data.

## Methods

In this article, we developed a framework for identifying the significant gene module from a RNA-seq expression dataset for a disease or specific cellular/physiological condition. The resultant gene modules will be integrated to obtain one final significant module. We used TCGA cervical cancer gene expression data and the phenotype data to test our method. The phenotype data was utilized to obtain the subtype of cervical cancer samples according to the Sample ID.

### Finding differentially expressed genes

First, we applied pre-filtering approaches (such as eliminating the genes having “all zeros”, “NA value removal”). After that, we carried out gene-wise standardization. Thereafter, Voom normalization [18] and Limma R tool [2, 47] were then utilized consecutively to identify the differentially expressed genes. Limma used empirical Bayes test. As a result, we obtained a set of statistically significant genes. After that, we applied volcano plot using bi-filtering approaches (*p*-value filtering and fold change filtering) consecutively. A up-regulated gene can be stated as a gene that had *p*-value less than 0.05 and fold change greater than 2, whereas a down-regulated gene be a gene having *p*-value less than 0.05 and fold change less than 0.5.

### Pareto-optimal cluster selection

Estimation of the Pareto-optimal cluster number of a data-profile is a challenging problem. Cluster validity indices are basically developed to evaluate the performance of a clustering and can be applied to rank various cluster sizes. After finding the set of the differentially expressed genes (up-regulated genes and down-regulated genes), we applied a R package MOCCA [27] on the data of the differentially expressed genes to determine the optimal (robust) number of clusters. Bootstrapping approach is utilized to identify the robust cluster numbers depending upon several cluster validity indices. Of note, these estimations will differ based on the employed clustering technique as well as the cluster validation index. The central idea of MOCCA approach is to evaluate the robust (Pareto-optimal) cluster numbers through aggregation of the best cluster numbers of various clustering algorithms and various cluster validation indices in a multi-objective environment. In details, firstly, MOCAA conducts a multi-objective optimization to accumulate cluster alternatives. Next, it extracts R number of bootstrap samples from data-matrix. It computes the clustering for all specific cluster numbers K through the use of three clustering techniques (kmeans, single-linkage and neuralgas clustering). Thereafter, it utilizes various cluster validation indices (MCA, Jaccard, FM and CQS) to the clustering. Hence, a total of twelve objective functions (i.e., kmeans.MCA, kmeans.Jaccard, kmeans.FM, kmeans.CQS, neuralgas.MCA, neuralgas.Jaccard, neuralgas.FM, neuralgas.CQS, single.MCA, single.Jaccard, single.FM and single.CQS) were provided here to obtain Pareto optimal (robust) number of clusters. However, these outcomes (cluster validity indices) were then compared through determining the Pareto-optimal cluster sizes and ranking them depending upon their domination. Finally, a vector having the rank of Pareto-optimal cluster sizes had been provided based upon their domination along with the matrix whose each row was connected with a specified Pareto-optimal cluster size, and each cell entry referred to as how many objective functions it dominates the clustering of the other remaining cluster sizes. Specially, the Pareto-optimal cluster sizes were ranked according to the lowest number of objectives for which they dominated other remaining cluster sizes.

After determining the optimal number of clusters, we identified the cluster information of each participated gene through k-means clustering using the optimal cluster size.

### Ranking of the clusters

After that, we computed Spearman’s Correlation Coefficient score among the participating pairwise genes belonging to each individual resultant cluster and determined the average Spearman’s Correlation Coefficient score of each cluster. The cluster whose average Spearman’s Correlation Coefficient score was maximum, was chosen as best cluster. The combined gene set of the best cluster was here treated as a gene-signature.

### Signature selection and validation through classifier

For the validation of the gene signature, we conducted a classification analysis containing all the features (genes) and all the samples having two groups (SCC and ADENO) using PAMR (prediction analysis for microarrays) tool [46].

Here, in details, we carried out 10-fold cross-validation and divided the data of all the genes belonging to the signature into training set and test set. Thereafter, we computed the threshold for which the error of the cross-validation would be minimum. PAMR classifier in “e107” R package [48] was then utilized using the training set and the resultant threshold for predicting the sample class of the test set. We repeat the whole process for 10 times. We used four evaluation metrics to compute the overall performance of the classification. Finally, we computed average sensitivity, average specificity, average precision and average accuracy.

**Fig. 1:**
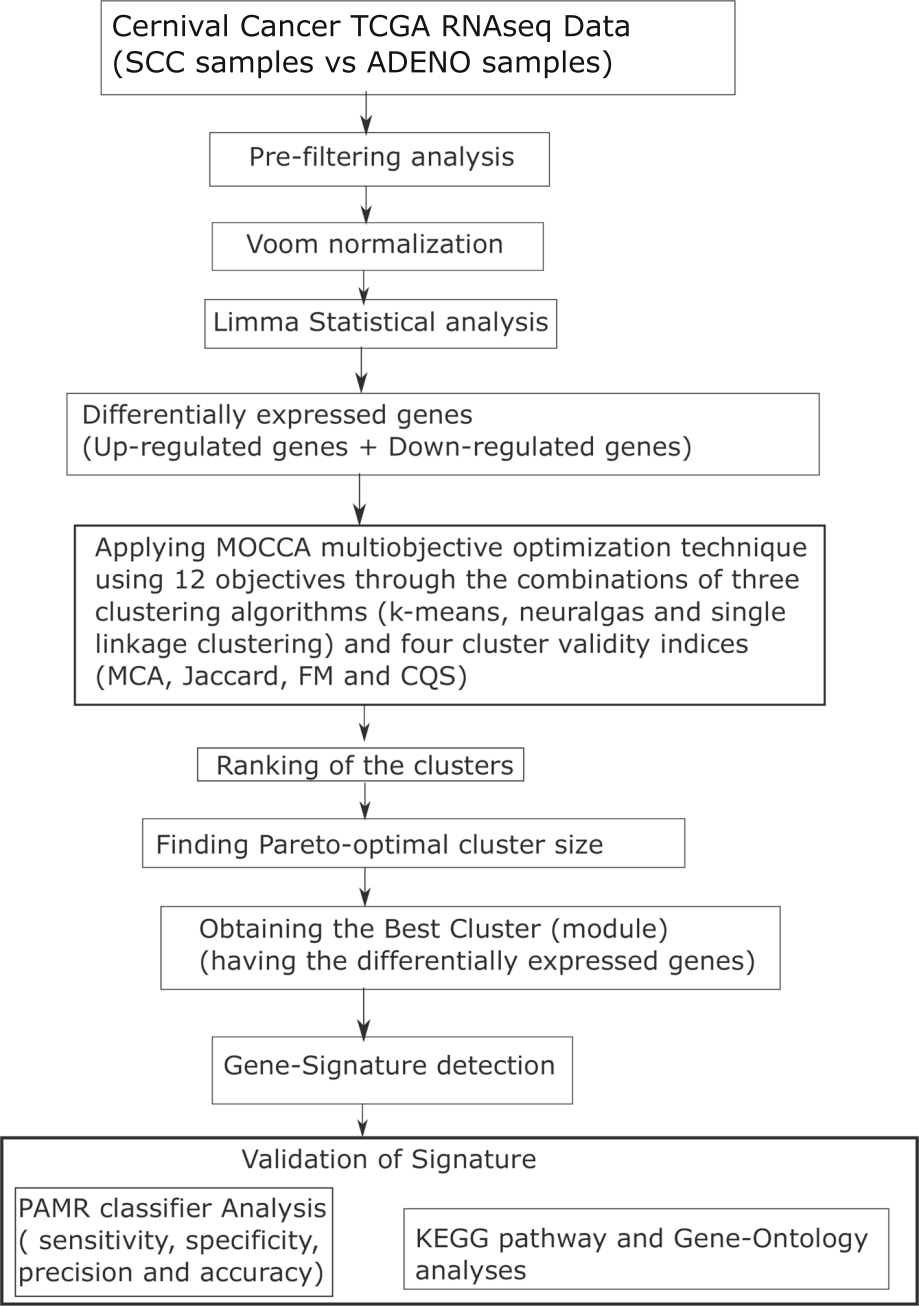
Flowchart of the proposed framework

In addition to the classifier design, we performed KEGG pathway and Gene-Ontology (GO) analyses for the participating genes of the signature using DAVID database. Here, we picked up the KEGG pathways or Gene-Ontology terms whose enrichment *p*-values were less than 0.05. Finally, we included a figure (Fig. 1) to represent all the steps of our method.

## Results

In this section, we firstly describe the source of the cervical cancer dataset and then demonstrate the experimental results.

We used TCGA Cervical Squamous Cell Carcinoma and Endocervical Adenocarcinoma (TCGA-CESC) gene expression data (IlluminaHiSeq platform) using UCSC Xena browser [49]. We used Endocervical type of adenocarcinoma (ADENO) as control sample and cervical squamous cell carcinoma (SCC) as diseased/experimental samples. The number of control samples (ADENO) is 22, whereas the number of experimental samples (SCC) is 253. The total number of genes is 20,530. We used these types of samples for comparison, the method can be applied to any pairs of samples (e.g. disease versus normal samples).

**Fig. 2:**
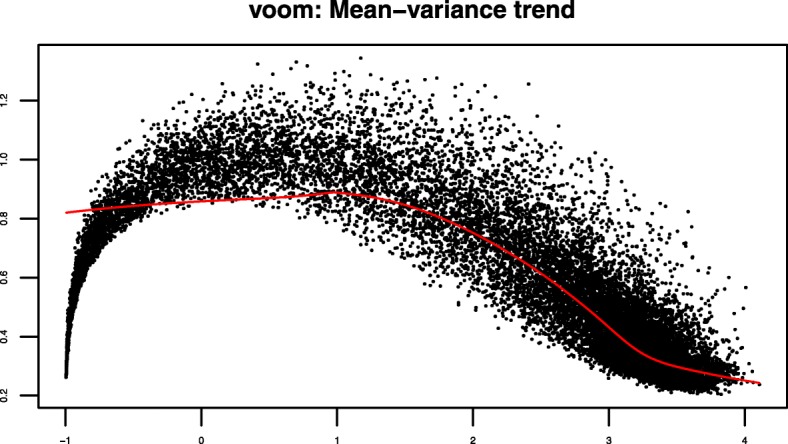
Voom normalization on the SCC and ADENO samples in TCGA cervical cancer dataset. SCC: squamous cell carcinoma. ADENO: adenocarcinoma

**Fig. 3:**
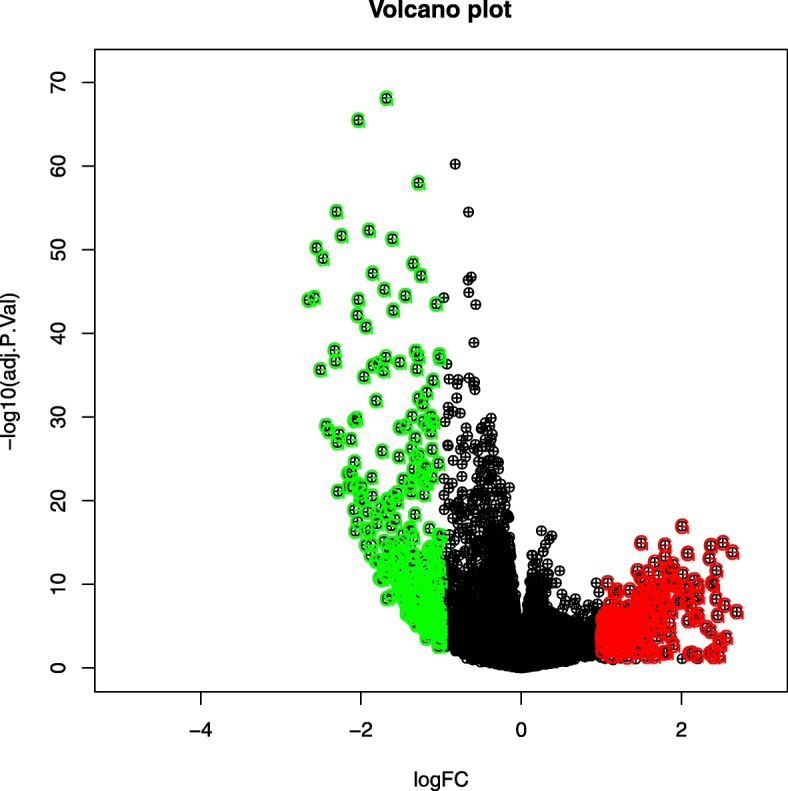
Volcano plot for identifying up-regulated and down-regulated genes for ADENO vs. SCC subtypes in TCGA cervical cancer dataset

First of all, we collected the data from the aforementioned TCGA cervical dataset covering a total of the 20,530 genes. We then eliminated the redundant features using subsequent pre-filtering approaches as mentioned in “Pareto-optimal cluster selection” section. After pre-filtering steps, we obtained a total of 19,709 genes. After that, we performed Voom normalization (in Fig. 2) and then Limma software consecutively to identify the differentially expressed genes. Thereafter, we utilized volcano plot (in Fig. 3) using bi-filtering approaches (*p*-value filtering and fold change filtering) consecutively. A up-regulated gene can be stated as a gene that had *p*-value less than 0.05 and fold change greater than 2, whereas a down-regulated gene be a gene having *p*-value less than 0.05 and fold change less than 0.5. As a result, we obtained a total number of 582 differentially expressed genes of which 260 are up-regulated genes and remaining 322 are down-regulated genes.

After finding the set of the up-regulated genes and down-regulated genes, we applied a R package MOCCA on the data of these genes to determine the Pareto-optimal (robust) number of cluster. As a result, we obtained 5 as Pareto-optimal cluster size. The objective values for the twelve objective functions are here represented in Table 1. After determining the optimal number of clusters (*n*=5), we collected the cluster information of each participated gene through k-means clustering using the optimal cluster size. Of note, the number of up-regulated genes in these resultant five clusters are 0, 4, 95, 35 and 126, respectively, whereas the number of down-regulated genes in these resultant five clusters are 113, 209, 0, 0 and 0, respectively. Of note, the modules might be detected in such an iteration of k-means clustering using the fixed number of cluster numbers when the genes were likely to be in convergence during the run of the k-means clustering. Here it might be possible to obtain such clusters (modules) having imbalanced number of upregulated genes and downregulated genes.

**Table 1:** Twelve objectives in MOCCA and their values from the TCGA cervical cancer RNA-seq dataset

Objective	Objective value
kmeans.MCA	0.602
kmeans.Jaccard	0.509
kmeans.FM	0.608
kmeans.CQS	0.978
neuralgas.MCA	0.602
neuralgas.Jaccard	0.518
neuralgas.FM	0.613
neuralgas.CQS	0.979
single.MCA	0.551
single.Jaccard	0.349
single.FM	0.520
single.CQS	0.977

**Fig. 4:**
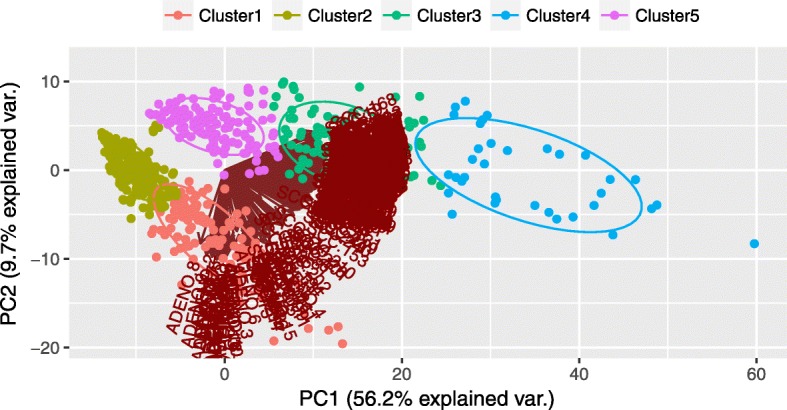
Principal Component Analysis (PCA) of the clustering genes obtained from the comparison of ADENO with SCC subtypes in TCGA cervical cancer dataset

Next, we calculated Spearman’s Correlation Coefficient score among the participating pairwise genes belonging to each individual resultant cluster, and determined the average Spearman’s Correlation Coefficient score of each cluster. The average Spearman’s Correlation of the five clusters were 0.312, 0.201, 0.309, 0.521 and 0.211, respectively. The fourth cluster having highest average Spearman’s Correlation Coefficient score (=0.521) was selected as “gene-signature”. Of note, the gene-signature contained 35 differentially expressed genes of which all the genes were up-regulated. These up-regulated genes are AKR1B10, ANXA8, ANXA8L2, BNC1, CLCA2, CSTA, DSC3, FBXO27, FOXE1, GBP6, GJB6, GPR109A, GPR115, GPR87, IVL, KRT6A, KRT6B, KRT6C, LOC642587, NCCRP1, PKP1, PLAC2, RHCG, SBSN, SERPINB13, SERPINB2, SERPINB4, SOX15, SPRR1A, SPRR2A, SPRR2D, TMEM40, TMPRSS11D, TP63 and VSNL1. Of note, we performed Principal Component Analysis (PCA) plot on the resultant genes belonging to the resultant clusters. The clusters are nicely visible in Fig. 4.

Thereafter, as mentioned in “Signature selection and validation through classifier” section, we performed 10-fold cross-validation and then ran PAMR classifier on all the features (genes) and all the samples having two groups (SCC and ADENO) belonging to the resultant signature. We repeat the whole process for 10 times. As a result, we obtained very good average accuracy (=93.45%(±0.297%)) in the classification study. For details about all the evaluation criteria (average sensitivity, average specificity, average precision, and average accuracy) are depicted in Table 2.

**Table 2:** Classification performance of the resultant gene signature having all the features and samples for the cervical cancer RNA-seq dataset

Evaluation criteria	Average(sd)
Sensitivity	0.934(0.27%)
Specificity	0.941(2.20%)
Precision	0.995(0.20%)
Accuracy	0.935(0.30%)

In addition, we performed gene set enrichment analysis using KEGG pathway and Gene-Ontology (GO) terms through DAVID online tool. As a result, we obtained one significant KEGG pathway (hsa05146:Amoebiasis having *p*-value 0.002) and nine significant GO:BP-terms. These GO terms are GO:0030216 keratinocyte differentiation (*p*-value 1.01x 10^−7^), GO:0018149 peptide cross-linking (*p*-value 9.91x 10^−7^), GO:0031424 keratinization (*p*-value 5.44x 10^−5^), GO:0008544 epidermis development (*p*-value 2.99x 10^−4^), GO:0010951 negative regulation of endopeptidase activity (*p*-value 8.41x 10^−4^), GO:0045104 intermediate filament cytoskeleton organization (*p*-value 0.023), GO:0010466 negative regulation of peptidase activity (*p*-value 0.026), GO:0030162 regulation of proteolysis (*p*-value 0.032) and GO:0031069 hair follicle morphogenesis (*p*-value 0.041). We also identified five significant GO:CC-terms. These GO terms are GO:0001533 cornified envelop (*p*-value 8.06x 10^−7^), GO:0070062 extracellular exosome (*p*-value 1.61x 10^−4^), GO:0045095 keratin filament (*p*-value 0.011), GO:0005882 intermediate filament (*p*-value 0.014) and GO:0030057 desmosome(*p*-value 0.038). We found five significant GO:MF-terms. These GO terms are GO:0005198 structural molecule activity (*p*-value 1.30x 10^−6^), GO:0030674 protein binding, bridging (*p*-value 0.006), GO:0004867 serine-type endopeptidase inhibitor activity (*p*-value 0.009), GO:0002020 protease binding (*p*-value 0.010) and GO:0004035 alkaline phosphatase activity (*p*-value 0.049). For details, see Table 3.

**Table 3:** KEGG pathway and Gene Ontology (GO) enrichment analysis of the participating genes of the resultant gene signature for the cervical cancer RNA-seq dataset

Gene set	*P*-value	Gene symbols
GO:BP ^*a*^: GO:0030216 keratinocyte differentiation	1.01x 10^−7^	*SPRR1A, SPRR2D, SPRR2A, TP63, CSTA, IVL*
GO:CC ^*b*^: GO:0001533 cornified envelop	8.06x 10^−7^	*SPRR1A, SPRR2D, SPRR2A, CSTA, IVL*
GO:BP: GO:0018149 peptide cross-linking	9.91x 10^−7^	*SPRR1A, SPRR2D, SPRR2A, CSTA, IVL*
GO:MF ^*c*^: GO:0005198 structural molecule activity	1.30x 10^−6^	*KRT6C, KRT6A, SPRR1A, SPRR2D, SPRR2A, CSTA, IVL*
GO:BP: GO:0031424 keratinization	5.44x 10^−5^	*SPRR1A, SPRR2D, SPRR2A, IVL*
GO:CC GO:0070062 extracellular exosome	1.61x 10^−4^	*KRT6C, KRT6A, GBP6, KRT6B, PKP1, NCCRP1, RHCG, TMPRSS11D, AKR1B10, SBSN, SERPINB4, SERPINB13, CSTA, IVL*
GO:BP: GO:0008544 epidermis development	2.99x 10^−4^	*SPRR1A, SPRR2D, SPRR2A, BNC1*
GO:BP: GO:0010951 negative regulation of endopeptidase activity	8.41x 10^−4^	*SERPINB2, SERPINB4, SERPINB13, CSTA*
KEGG pathway: hsa05146:Amoebiasis	0.002	*SERPINB2, SERPINB4, SERPINB13*
GO:MF: GO:0030674 protein binding, bridging	0.006	*SPRR1A, CSTA, IVL*
GO:MF GO:0004867 serine-type endopeptidase inhibitor activity	0.009	*SERPINB2, SERPINB4, SERPINB13*
GO:MF: GO:0002020 protease binding	0.010	*SERPINB4, SERPINB13, CSTA*
GO:CC: GO:0045095 keratin filament	0.011	*KRT6C, KRT6A, KRT6B*
GO:CC: GO:0005882 intermediate filament	0.014	*KRT6C, KRT6A, PKP1*
GO:BP: GO:0045104 intermediate filament cytoskeleton organization	0.023	*KRT6C, KRT6A*
GO:BP: GO:0010466 negative regulation of peptidase activity	0.026	*SERPINB4, CSTA*
GO:BP: GO:0030162 regulation of proteolysis	0.032	*SERPINB4, SERPINB13*
GO:CC: GO:0030057 desmosome	0.038	*PKP1, DSC3*
GO:BP: GO:0031069 hair follicle morphogenesis	0.041	*FOXE1, TP63*
GO:MF: GO:0004869 cysteine-type endopeptidase inhibitor activity	0.049	*SERPINB13, CSTA*

^*a*^ Biological Processing, ^*b*^ Cellular Components, ^*c*^ Molecular Function

## Discussion

There are a lot of group lasso techniques (sglasso [50], flasso [50], etc.) available in the literature. But the objectives of these lasso techniques are different from our method. Lasso technique is basically a regression based study, whereas our method is Pareto optimal based clustering framework used only a single genomic or epigenetic data. So, we cannot compare our method with lasso based approaches. Furthermore, in the literature, there are a lot of co-expression based techniques for gene signature identification. But, the majority of the existing methods either follow a WGCNA module detection method or something like that where the generalized modules are not optimized [51, 52]. If the input threshold for the minimum number of module is changed, the number of modules is likely to change. To recover from the method, we first optimized the number of clusters in our method, and then used a standard clustering algorithm to find gene modules. Finally, we computed average Spearman’s Correlation Coefficient of each module, and obtained the top ranked module as gene signature. Moreover, our method produces very high classification performance for the signature in terms of sensitivity, specificity, accuracy and precision. Hence, our method is beneficial in various aspects rather than other related existing methods.

## Conclusions

Although there are many bioinformatics approaches available for the gene signature identification, the gene signature identification through Pareto-optimal technique has never been tried before. Therefore, in this article, we developed a new framework of identifying gene signature using Pareto-optimal cluster identification for RNA-seq data. In this regard, we conducted some pre-filtering steps to remove the redundant feature from the dataset. Next, we applied Voom normalization and then Limma statistical tool to find the differentially expressed genes consisting of up-regulated and down-regulated genes. Thereafter, we applied MOCCA R tool on these differentially expressed genes to estimate the Patero-optimal cluster size, and then applied k-means clustering through the optimal cluster size. The best cluster was obtained through computing the average Spearman’s Correlation Score among all the genes in pair-wise manner belonging to the module/cluster. The best cluster is now treated as a signature for the respective disease. For validation, we applied PAMR classifier on all the genes of selected best cluster, and computed the classification performances. In this work, we used TCGA cervical cancer dataset for testing, and we found a 35 gene signature. We obtained high average classification accuracy (=0.935(±0.297%)). The signature might be helpful for diagnosis of the disease. Finally, our method is useful to identify gene signature for any RNA-seq or similar kind of data.

## Abbreviations

ADENO: Adenocarcinoma
BP: Biological processing
CC: Cellular component
CESC: Cervical squamous cell carcinoma and endocervical adenocarcinoma
DAVID: Database for annotation, visualization and integrated discovery
DEG: Differentially expressed genes
FM: F measure
GO: Gene-ontology
KEGG: Kyoto encyclopedia of genes and genomes
Limma: Linear models for microarray data
MCA: Multiple correspondence analysis
MF: Molecular function
MOCCA: Multi-objective optimization for collecting cluster alternatives
PAM: Prediction analysis for microarrays
PCA: Principal component analysis
SCC: Squamous cell carcinoma
WGCNA: Weighted correlation network analysis
